# The *BDNF* p.Val66Met polymorphism, childhood trauma, and brain volumes in adolescents with alcohol abuse

**DOI:** 10.1186/s12888-014-0328-2

**Published:** 2014-12-16

**Authors:** Shareefa Dalvie, Dan J Stein, Karestan Koenen, Valerie Cardenas, Natalie L Cuzen, Raj Ramesar, George Fein, Samantha J Brooks

**Affiliations:** MRC/UCT Human Genetics Research Unit, Division of Human Genetics, Institute of Infectious Disease and Molecular Medicine, Faculty of Health Sciences, University of Cape Town, Observatory, 7925 Cape Town South Africa; Department of Psychiatry and Mental Health, University of Cape Town, Observatory, Cape Town South Africa; Mailman School of Public Health, Columbia University, New York, NY USA; Neurobehavioral Research Inc, Honolulu, HI USA

**Keywords:** Childhood trauma questionnaire, Alcohol use disorders, Magnetic resonance imaging, Voxel-based morphometry

## Abstract

**Background:**

Previous studies have indicated that early life adversity, genetic factors and alcohol dependence are associated with reduced brain volume in adolescents. However, data on the interactive effects of early life adversity, genetic factors (e.g. p.Met66 allele of *BDNF*), and alcohol dependence, on brain structure in adolescents is limited. We examined whether the *BDNF* p.Val66Met polymorphism interacts with childhood trauma to predict alterations in brain volume in adolescents with alcohol use disorders (AUDs).

**Methods:**

We examined 160 participants (80 adolescents with DSM-IV AUD and 80 age- and gender-matched controls) who were assessed for trauma using the Childhood Trauma Questionnaire (CTQ). Magnetic resonance images were acquired for a subset of the cohort (58 AUD and 58 controls) and volumes of global and regional structures were estimated using voxel-based morphometry (VBM). Samples were genotyped for the p.Val66Met polymorphism using the TaqMan® Assay. Analysis of covariance (ANCOVA) and post-hoc t-tests were conducted using SPM8 VBM.

**Results:**

No significant associations, corrected for multiple comparisons, were found between the *BDNF* p.Val66Met polymorphism, brain volumes and AUD in adolescents with childhood trauma.

**Conclusions:**

These preliminary findings suggest that the *BDNF* p.Met66 allele and childhood trauma may not be associated with reduced structural volumes in AUD. Other genetic contributors should be investigated in future studies.

## Background

Early brain development is strongly influenced by a range of environmental factors, including exposure to substances such as alcohol [1], as well as exposure to traumas such as early adversity, abuse and neglect [2]. Neuroimaging studies have indicated that various brain regions are altered in those who are alcohol dependent. In comparison to healthy controls, individuals who are alcohol dependent have smaller prefrontal cortical volumes [3], right hippocampal volumes [4,5], amygdala [6] and grey and white matter volumes [7,8]. Studies of alcohol exposure in adolescents have, however, been confounded by drug and psychiatric comorbidity. In contrast to studies in adults, we found that in adolescents with alcohol exposure but with no comorbidity, the pattern of grey matter density differences compared to controls was limited to regions in the left lateral frontal, parietal and temporal lobes [8].

Studies of exposure to childhood trauma have been associated with reduced brain volumes in the prefrontal cortex [9] and the hippocampus [10-12]. This is consistent with animal studies, which demonstrated that rats exposed to early life stress have alterations in hippocampal volume, possibly as a result of a decreased rate of synaptic development [13]. A more recent study found that early life stress was associated with changes in neuronal firing rates in the hippocampal and thalamocortical brain regions in rats [14]. In a further analysis of our data on adolescents, we found that childhood trauma is associated with smaller left hippocampal and right precentral gyrus volumes in adolescents with exposure to alcohol [15].

Brain development is, however, influenced not only by exposure to alcohol and childhood stressors, but also by genetic factors. Twin studies have shown that brain structure has considerable heritability [16-18]. However, exactly which genes are responsible for brain volume variation is not yet clarified. A strong candidate is the *brain derived neurotrophic factor* (*BDNF*) gene (chr11p13) which has previously been associated with variation in brain volumes [19-21]. Of particular interest is the p.Val66Met functional polymorphism, which is characterized by a valine to methionine substitution at codon 66 and is located in the pro-domain of the protein [22]. Although the pro-domain is cleaved from the mature protein, it is thought to be involved in the trafficking of BDNF and specifically, p.Met66 allele carriers (heterozygous p.Val66Met and homozygous p.Met66) have lower serum BDNF levels [23], as well as smaller hippocampal and prefrontal cortical volumes [19] compared to individuals who are homozygous for the p.Val66 allele.

We hypothesized that neural development during adolescence is jointly influenced by genetic and environmental factors, such as exposure to childhood trauma or excess alcohol use. For example, those with the *BDNF* p.Met66 allele might be more vulnerable to the effects of early life adversity and alcohol use. Based on our previous research we have found that left lateral frontal, parietal and temporal lobes as well as hippocampal volumes may be associated with adolescent alcoholism, childhood trauma, or both [8,15]. However, it is not yet known whether the *BDNF* p.Val66Met polymorphism interacts with brain volume in these regions. Therefore, the aim of this investigation was to determine whether the *BDNF* p.Val66Met polymorphism interacts with levels of childhood trauma, and alcohol use disorders (AUD) to result in alterations in brain volume in a cohort of adolescents.

## Methods

### Participants

Ethical approval for this study was obtained from the Research Ethics Committees of Stellenbosch University (N06/07/128) and the University of Cape Town (HREC REF 023/2012). Detailed accounts of the procedures involved in participant recruitment are outlined in previous studies [8,24]. Briefly, subjects were English- and Afrikaans-speaking adolescents from 19 schools within the Cape Flats region (within a 30 km radius of the test site at Tygerberg Hospital) of the greater Cape Town metropole and were from moderately low socioeconomic backgrounds. Participants were recruited to the study by means of oral presentations at schools and advertisement via word-of-mouth. Individuals who consumed alcohol on a regular basis and those who did not consume alcohol were invited to participate in the study. Convenience sampling procedures were used. Alcohol users were selected on a first come basis from the 890 volunteers who expressed interest in being included in the study. Controls were matched for each of these individuals based on similarity of sociodemographic profile (i.e. age within 1 year; same sex, language, ethnicity, and socioeconomic status). Volunteers not meeting eligibility criteria for possible inclusion in the alcohol or control groups were excluded at the prescreening stage.

Exclusion criteria for study participation were intellectual disability, lifetime DSM-IV Axis I diagnosis other than AUD, current use of sedative or psychotropic medication, signs or history of fetal alcohol syndrome or malnutrition, sensory impairment, history of traumatic brain injury, presence of diseases that may affect the CNS (e.g. meningitis, epilepsy, HIV), less than 6 years of formal education, and lack of proficiency in English or Afrikaans. An additional exclusion criterion was lifetime dosages exceeding 30 cannabis joints or 3 methamphetamine doses*.* A previous study defined significant marijuana use as greater than 100 episodes [25] and another study investigated adolescent methamphetamine and cannabis users that had an average of 1099 lifetime cannabis doses and 579 (methamphetamine only) and 837 (methamphetamine and cannabis) doses [26]. Our cohort is well below those limits so we can conclude that our subjects were not significant cannabis or methamphetamine users. The final cohort consisted of 80 adolescents with DSM-IV AUD and 80 age- and gender-matched light/non-drinking controls (HC). Blood samples were collected from each participant with the appropriate written informed assent and written informed consent was obtained from their parents or guardians.

### Measures

#### Early adversity

Childhood adversity was measured by the 28 item Childhood Trauma Questionnaire- Short Form (CTQ-SF) [27]. This self-report questionnaire consists of five subscales, each measuring a specific dimension of childhood maltreatment: physical abuse, sexual abuse, emotional abuse, physical neglect and emotional neglect. Each dimension consists of 5 items [27].

### Alcohol use

To determine current and past psychiatric diagnoses, each of the participants was interviewed with the Schedule for Affective Disorders and Schizophrenia for school-aged children (6–18 years) Lifetime Version (K-SADS-PL) [28]. In addition to the K-SADS-PL, the Timeline Followback (TLFB) procedure was used to determine lifetime history of alcohol use and drinking patterns [29].

### Neuroimaging

#### MRI acquisition

Magnetic resonance images (MRIs) were collected for a subset of the cohort (58 AUD and 58 HC) with a 3 T Siemens Magnetom Allegra MR Headscanner using Syngo MR software (Siemens Medical Solutions). The scanner is located in the Cape Universities Brain Imaging Center at the Stellenbosch University Health Sciences Campus, South Africa. Images for 50 subjects (25 HC and 25 AUD) were acquired using a transaxial T1-weighted acquisition (TR =2080 ms, TE =4.88 mm, acquisition matrix =256 x 192) at 1.0 mm thickness. The initial review of these images revealed undesirable presence of blood-vessels in the imaging, resulting from the fact the scanner used is a head-only model. This does not allow proper saturation of the blood to suppress its signal before it enters the head. To reduce the signal from unsaturated blood, the use of a sagittal T1 protocol was instituted (TR =2200 ms, TE =5.16 ms, acquisition matrix 256 × 256) at 1.0 mm thickness. A total of 66 individuals (33 HC and 33 AUD) had an MRI using the sagittal protocol only. Of the 50 individuals with a transaxial T1-weighted acquisition, 25 individuals (9 HC and 16 AUD) had an additional MRI with the sagittal protocol. In prior work we demonstrated that the two acquisition protocols produced comparable images that could be combined for analysis [8], although out of an abundance of caution we match the HC and AUD subjects on imaging protocol in imaging analyses reported in this paper.

### MRI analysis

After manually reorienting and realigning the cross-hair on the AC-PC plane in all our nifti-converted DICOM T1 images, and imposing initial quality control for signal artifacts, morphological changes were calculated using the voxel-based morphometry (VBM) default unified segmentation approach [30] in SPM8 (http://www.fil.ion.ucl.ac.uk/spm/software/spm8/). First we co-registered the T1 images to the Cincinnati Children’s Hospital global old children template (www. irc.cchmc.org/software/pedbrain.php). Using the old children templates for grey matter, white matter and cerebrospinal fluid, our images were then classified under the segmentation step as grey matter (GM), white matter (WM), and cerebrospinal fluid (CSF) and maps of GM probability at each voxel were derived. The GM probability maps were spatially normalized and co-registered using the same segmented template. The probability maps of gray matter were “modulated” to account for the effect of spatial normalisation, by multiplying the probability value of each voxel by its relative volume in native space before and after warping. Modulated images were smoothed with an 8 mm ‘Full Width Half Maximum [FWHM]’ Gaussian kernel, in line with other recent VBM studies. This smoothing kernel was applied prior to statistical analysis, to reduce signal noise and to correct for image variability unaccounted for by spatial normalization. All subsequent VBM analyses were corrected for total matter volume (TMV), representing total GM and WM but not CSF, as there is some evidence that CSF is over-estimated by SPM8 [31].

### Genotyping

DNA from subjects and controls was extracted using the Maxwell® 16 Blood DNA purification kit (AS1010) (Promega) using the Maxwell 16 instrument (Promega) at the Centre for Proteomic and Genomic Research (CPGR) (Cape Town, South Africa). Genotyping was performed using the TaqMan® SNP Genotyping Assay, which was run on the AB7900HT qPCR instrument (Life Technologies) at the CPGR (Cape Town, South Africa). Assay quality control measures included a no template control and a positive control. Genotyping data was validated using an Illumina Infinium iSelect custom 6000 bead chip which was run on Illumina’s BeadStation 500G Systems at the University of Michigan DNA Sequencing Core (Michigan, USA).

### Statistical analysis

The Shapiro-Wilk test was used to determine whether the continuous variables were normally distributed. The Mann Whitney U-test was performed to determine whether there were differences in these variables between the genders and the groups (HC vs AUD). Genotype by group (AUD vs HC) association calculations were performed using logistic regression using the total cohort of 80 HC and 80 AUD. All of the above mentioned tests were carried out using the statistical program SPSS [32]. Using the Exact test, deviation from Hardy-Weinberg Equilibrium (HWE) was calculated using the *genetics* package in the statistical environment R (http://r.adu.org.za/).

In the smaller imaging cohort of 58 HC and 58 AUD, to examine the main effects of group (AUD and HC), CTQ score (high vs low), genotype (homozygous p.Val66 vs. p.Val66Met and homozygous p.Met66) and the interaction between genotype and CTQ on brain volume data, 2 × 2 ANCOVA using VBM in the SPM8 package (http:http://www.fil.ion.ucl.ac.uk/spm/software/spm8/) was implemented. Based on our previous findings, region of interest (ROI) analyses were derived from templates in the aal atlas, representing the left lateral frontal, parietal and temporal lobes as well as hippocampal volumes [8,15]. AUD and HC subjects were matched in terms of age, gender, and protocol. Age and TMV was added as covariates of no interest to control for global differences in head size and emphasise local volume differences, and total CTQ score was included as a covariate of interest. All statistical analyses were corrected for multiple comparisons at the peak voxel level using the family-wise error (FWE).

## Results

### Demographics, alcohol use, trauma and genotype information

Sociodemographic and clinical data for the cohort are presented in Table 1. As expected, adolescents with AUDs had significantly higher lifetime doses of alcohol than the HC group (Mann Whitney U-test *p*-value < 0.001; Table 2). In the total cohort, the median total CTQ score was 36.50 and 42.00 for the HC and AUD group, respectively. This difference in median total CTQ scores was statistically significant (Mann Whitney U-test *p*-value = 0.023) (see Table 2 for further details, including the subscale scores). The median ages of the HC and AUD groups for the total cohort were 14.92 and 14.94, respectively (Table 1). The majority of the study participants were Afrikaans speaking and the median number of years of education was 8.0 years for both groups (HC and AUD). The median number of alcohol life dose units for the AUD group in the total cohort was 1125.50, where a unit refers to one beer or wine cooler, one glass of wine, or one 43 g shot of liquor (on its own, or in a mixed drink).

**Table 1:** **Median values and interquartile range for cohort characteristics**

**Variable**	**HC**			**AUD**		
**Total cohort**	**Females (** ***n*** **= 47)**	**Males (** ***n*** **= 33)**	**Total (** ***n*** **= 80)**	**Females (** ***n*** **= 47)**	**Males (** ***n*** **= 33)**	**Total (** ***n*** **= 80)**
*Imaging cohort* ^*b*^	*Females (n = 33)*	*Males (n = 25)*	*Total (n = 58)*	*Females (n = 33)*	*Males (n = 25)*	*Total (n = 58)*
Age (years)	14.89 (15.32-14.4)	14.94 (15.45-14.23)	14.92 (15.33-14.36)	14.89 (15.59-14.31)	14.98 (15.51-14.52)	14.94 (15.53-14.47)
	*14.71 (15.14-14.35)*	*15.07 (15.54-14.32)*	*14.77 (15.33-14.35)*	*14.89 (15.64-14.06)*	*15.17 (15.57-14.52)*	*14.98 (15.60-14.41)*
Education (years)	8.0 (8.0-7.0)	8.0 (8.0-7.0)	8.0 (8.0-7.0)	8.0 (8.0-7.0)	8.0 (8.0-7.0)	8.0 (8.0-7.0)
	*8.0 (8.0-7.0)*	*8.0 (8.0-7.0)*	*8.0 (8.0-7.0)*	*8.0 (9.0-7.0)*	*8.0 (8.0-7.0)*	*8.0 (8.0-7.0)*
% Afrikaans-speaking	63.8	78.8	70.0	63.8	75.8	68.8
	*69.7*	*84*	*75.9*	*60.6*	*72.0*	*65.5*
Alcohol life dose units^a^	1.0 (9.00-0)	1.0 (4.0-0)	1.0 (5.75-0.0)	1152.0 (2008.0-480.0)	1012.0 (2076.0-396.0)	1125.50 (2032.0-441.0)
	*1.0 (9.00-0)*	*1.0 (3.5-0)*	*1.0 (4.25-0.0)*	*892.0 (1980.0-442.0)*	*1012.0 (2076.0-396.0)*	*962.0 (1987.0-429.0)*
CTQ-Physical Abuse	5.0 (7.0-5.0)	5.0 (6.0-5.0)	5.0 (7.0-5.0)	5.0 (7.0-5.0)	7.0 (10.0-5.0)	5.0 (7.0-5.0)
	*5.0 (6.5-5.0)*	*5.0 (6.0-5.0)*	*5.0 (6.0-5.0)*	*5.0 (7.0-5.0)*	*7.0 (12.0-5.0)*	*6.0 (7.5-5.0)*
CTQ-Sexual abuse	5.0 (5.0-5.0)	5.0 (5.0-5.0)	5.0 (5.0-5.0)	5.0 (6.0-5.0)	6.0 (9.0-5.0)	5.0 (7.0-5.0)
	*5.0 (5.0-5.0)*	*5.0 (5.0-5.0)*	*5.0 (5.0-5.0)*	*5.0 (5.0-5.0)*	*6.0 (12.0-5.0)*	*5.0 (7.0-5.0)*
CTQ-Emotional Abuse	6.0 (9.0-5.0)	6.0 (8.0-5.0)	6.0 (8.75-5.0)	8.0 (11.0-6.0)	9.0 (11.5-5.0)	8.0 (11.0-6.0)
	*5.0 (8.5-5.0)*	*6.0 (8.5-5.0)*	*6.0 (8.25-5.0)*	*8.0 (11.0-6.0)*	*9.0 (11.5-5.0)*	*8.0 (11.0-6.0)*
CTQ-Physical Neglect	6.0 (9.0-5.0)	8.0 (10.5-5.0)	7.0 (9.0-5.0)	9.0 (11.0-7.0)	8.0 (13.0-5.5)	8.50 (12.0-6.0)
	*5.0 (9.0-5.0)*	*7.0 (12.0-5.5)*	*7.0 (9.25-5.0)*	*8.0 (11.0-6.5)*	*9.0 (12.5-5.5)*	*8.0 (12.0-6.0)*
CTQ-Emotional Neglect	10.0 (13.0-6.0)	11.0 (18.5-8.0)	11.0 (15.0-7.0)	12.0 (17.0-7.0)	12.0 (19.0-8.0)	12.0 (18.0-8.0)
	*10.0 (13.0-6.0)*	*13.0 (21.0-9.0)*	*11.0 (17.0-7.75)*	*12.0 (17.5-7.5)*	*12.0 (17.0-8.0)*	*12.0 (17.25-8.0)*
CTQ-total score	35.0 (46.0-29.0)	39.0 (47.0-30.5)	36.50 (46.0-29.25)	42.0 (51.0-34.0)	42.0 (60.0-30.0)	42.00 (53.5-33.25)
	*35.0 (46.0-29.0)*	*39.0 (52.0-30.5)*	*36.0 (49.0-29.75)*	*42.0 (48.0-34.5)*	*42.0 (65.5-33.0)*	*42.0 (52.0-34.75)*

^a^Alcohol life dose was measured in units. One unit was defined as one beer or wine cooler, one glass of wine, or one 43 g shot of liquor (alone or in a mixed drink). ^b^Imaging cohort values are italicized.

**Table 2:** **Comparison of cohort characteristics for HC and AUD groups-** ***p*** **-values from Mann Whitney U-test**

	**HC**	**AUD**	**HC vs AUD**		
	**Females vs males**	**Females vs males**	**Females**	**Males**	**Total**
Age (years)	0.949	0.581	0.589	0.401	0.313
	^*d*^ *0.162*	*0.388*	*0.254*	*0.535*	*0.159*
Education (years)	0.132	0.646	0.517	0.192	0.751
	*0.689*	*0.729*	*0.706*	*0.671*	*0.577*
% Afrikaans-speaking^c^	0.151	0.257	1.000	0.769	0.864
		*0.366*	*0.438*	*0.306*	*0.221*
Alcohol life dose units	0.385	0.503	<0.001	<0.001	<0.001
	*0.538*	*0.881*	*<0.001*	*<0.001*	*<0.001*
CTQ-Physical Abuse	0.561	0.023	0.983	0.018	0.116
	*0.807*	*0.008*	*0.678*	*0.008*	*0.031*
CTQ-Sexual abuse	0.455	0.018	0.398	0.001	0.004
	*0.729*	*0.036*	*0.615*	*0.011*	*0.030*
CTQ-Emotional Abuse	0.806	0.640	0.025	0.074	0.004
	*0.967*	*0.950*	*0.034*	*0.063*	*0.005*
CTQ-Physical Neglect	0.199	0.984	0.014	0.605	0.032
	*0.084*	*0.658*	*0.025*	*0.747*	*0.074*
CTQ-Emotional Neglect	0.109	0.984	0.128	0.686	0.410
	*0.072*	*0.747*	*0.226*	*0.289*	*0.881*
CTQ-total score	0.384	0.443	0.048	0.162	0.023
	*0.203*	*0.362*	*0.080*	*0.268*	*0.062*

^c^Pearson chi-squared test (*d.f*. =1).^d^Imaging cohort values are indicated in italics.

The genotype frequencies in the total cohort (80 AUD and 80 HC) were homozygous p.Val66 = 0.79, heterozygous p.Val66Met =0.15 and homozygous p.Met66 = 0.06 for cases and homozygous p.Val66 = 0.83, heterozygous p.Val66Met =0.16 and homozygous p.Met66 = 0.01 for controls. The genotype frequencies in the imaging cohort (58 AUD and 58 HC) were homozygous p.Val66 = 0.76, heterozygous p.Val66Met =0.17 and homozygous p.Met66 = 0.07 for cases and homozygous p.Val66 = 0.78, heterozygous p.Val66Met =0.21 and homozygous p.Met66 = 0.02 for controls. As there were a small number of individuals with the p.Met66 allele, heterozygous individuals were grouped with individuals homozygous for the p.Met66 allele. There was no significant association between the p.Val66Met polymorphism and AUD when comparing all genotype groups (*p*-value = 0.323) independently and when grouping the heterozygous p.Val66Met and homozygous p.Met66 genotypes together (*p*-value = 0.549). For the HC group, this polymorphism was in HWE (*p*-value =0.51). Besides CTQ-emotional abuse (*p*-value = 0.032), none of the sociodemographic or CTQ scores differed between the homozygous p.Val66 group and the p.Met66 (homozygous and heterozygous) group.

### Voxel-based morphometry

We conducted a 2 × 2 ANCOVA of brain volume differences for group (AUD, HC) and BDNF status (homozygous p.Val66 vs. heterozygous p.Val66Met and homozygous p.Met66 allele), using VBM and included CTQ total score as a covariate. We did not find any statistically significant associations between the *BDNF* p.Val66Met polymorphism, structural brain volumes and AUD, for main effects or as an interaction. Our strongest findings, uncorrected for multiple comparisons and therefore preliminary, was found in the left lateral prefrontal cortex (PFC) (Table 3). Uncorrected main effects of AUD status and genotype were observed in the left lateral PFC (x = −43, y = 48, z = −17, p < 0.001), respectively. Specifically, individuals in the HC group, homozygous for the p.Val66 allele, had larger left lateral PFC volumes than individuals in the AUD group with the same genotype. When only examining the AUD group, smaller left lateral PFC volume (x = −38, y = 13, z = 28, p < 0.001) was observed in individuals with the p.Met66 allele.

**Table 3:** **2 × 2 ANCOVA (group x genotype) matched for age, gender and protocol**

	**MNI** ^**e**^ **Coordinates**						
**Brain region**	***x***	**y**	**z**	**Brodmann Area**	**Cluster Size (Voxels)**	**Z-statistic**	**Cluster p-value**
**Ancova analyses:**							
*Main effect of group*							
Left lateral prefrontal cortex	−43	48	−17	11	125	3.64	<0.001
*Main effect of genotype*							
Left lateral prefrontal cortex	−43	48	−17	11	129	3.66	<0.001
Left occipital lobe	−32	−88	3	19	58	3.52	<0.001
*Main effect of CTQ total score*							
Left parahippocampal gyrus	−26	−24	−25	35	958	4.16	<0.001
*Genotype x alcohol group interaction*							
Left lateral prefrontal cortex	−43	48	−17	11	32	3.33	<0.001
*CTQ total score x genotype interaction*							
Fusiform gyrus	39	−59	−21	NA^f^	890	3.69	<0.001
**POST-HOC t-tests** ^**g**^ **:**							
*Genotype post-hoc t-test: Val/Val > Val/Met + Met/Met*							
Left lateral prefrontal cortex	−40	12	28	9	29	3.24	<0.001
*Within-group genotype t-test (AUD): Val/Val > Val/Met + Met/Met*							
Left lateral prefrontal cortex	−38	13	28	9	17	3.18	<0.001
*Between-group genotype t-test (Val/Val): HC > AUD*							
Left lateral prefrontal cortex	−20	23	−30	11	332	3.88	<0.001
*Within-group CTQ t-test ( Val/Met + Met/Met ): low > high CTQ*							
Middle Occipital gyrus	−17	−93	14	18	708	4.78	<0.001
*Within-group CTQ t-test (Val/Val): low > high CTQ*							
Middle frontal gyrus	−27	25	−18	NA	708	4.06	<0.001

^e^MNI = Montreal Neurological Institute CoordinateSs. ^f^Not applicable. ^g^Besides the CTQ t-tests, all post-hoc t-tests were analysed with CTQ as a covariate of interest.

As an additional 2 × 2 ANCOVA, we examined CTQ as a main effect and as an interaction with *BDNF* genotype, controlling for age, TMV and alcohol life dose as covariates. Thus, we examined high vs. low CTQ, dichotomized by percentile, and *BDNF* dichotomized by homozygous p.Val66 vs. heterozygous p.Val66Met and homozygous p.Met66. Using this approach an uncorrected main effect of CTQ was observed in the left parahippocampal gyrus (x = −26, y = −24 z = −25, p < 0.001). An uncorrected interaction was shown between CTQ and *BDNF* genotype in the fusiform gyrus (x = 39, y = −59 z = −21, p < 0.001). Post-hoc t-tests (uncorrected) revealed smaller volumes in the middle occipital gyrus (x = −17, y = −93 z = 14, p < 0.001) and middle frontal gyrus (x = −27, y = 25 z = −18, p < 0.001), in individuals with high CTQ total scores compared to those with low scores, in individuals with the p.Met66 allele and those homozygous for the p.Val66 allele, respectively (Table 3).

## Discussion

This investigation sought to determine whether the *BDNF* p.Val66Met is associated with brain volume alterations in a cohort of adolescents with AUD and who have experienced childhood trauma. In a previous analysis of structural brain volume in adolescents with AUD, AUD was associated with reduced brain volumes in left lateral frontal, temporal and parietal regions [8]. Additionally, in a study conducted by our group, we controlled for childhood trauma in an adolescent AUD cohort [15], and found reduced frontal and temporal cortices and hippocampal volumes. Here we attempted to extend previous work by examining whether the *BDNF* p.Val66Met polymorphism, AUD and childhood trauma jointly affect brain volume in adolescents. We were unable to find any significant main effects or interaction between the *BDNF* p.Val66Met, levels of childhood trauma, and AUD resulting in structural brain volume differences. This is consistent with previous studies which did not find an effect of this polymorphism on brain volumes and AUD [33-36].

As part of a secondary exploratory analysis, when examining findings uncorrected for multiple comparisons, the *BDNF* p.Met66 allele was associated with smaller lateral PFC volume, an area involved with behavioral executive functioning [37]. Similarly, previous studies have shown an association between the *BDNF* p.Met66 allele and reduced dorsolateral PFC (DLPFC) volumes [19,38]. This preliminary and uncorrected finding is also in accordance with a previous study which found that childhood emotional neglect was associated with reduced DLPFC [39]. Most studies report associations between the p.Met66 allele and decreases in hippocampal volume [19,20,40,41]. This association between the p.Met66 allele and hippocampal volume is also observed in individuals with high levels of childhood trauma [42]. However, it has been proposed that adverse hippocampal development also significantly impinges on DLPFC development [43], as there is some evidence that these regions are functionally connected [44]. Thus, having the *BDNF* p.Val66Met polymorphism may interact negatively with the experience of childhood trauma, which may in turn affect the development of the hippocampus and the DLPFC [45].

One of the limitations of this study is the small sample size. An increase in sample size may effectively increase the power of the study and enable the detection of findings that are corrected for multiple comparisons. Certainly, studies with larger cohorts are needed to confirm our preliminary interpretations of the first exploration into how the *BDNF* p.Val66Met genotype and early life adversity interact with brain volumes in adolescents with AUD. Also, only one polymorphism within the *BDNF* gene was investigated. To gain a broader understanding of the role of *BDNF* in brain volume variation, AUD and childhood adversity, variation (in linkage equilibrium) across the entire gene should be considered as well as the expression of the gene in brain volumes of interest. As the entire cohort in this study consisted of adolescents of mixed ancestry, ethnicity was not added as a covariate in the analysis. However, the mixed ancestry group is an admixed population; therefore the analysis could have been influenced by population stratification. Furthermore, no information was obtained regarding the familial history of AUDs in our subjects, a possible confounder. Previous research has shown that a positive family history of alcohol use has an association with increased alcohol consumption in college students [46]. Another limitation is the fact that brain images were obtained using different imaging protocols. However, we attempted to overcome this by matching scans based on protocol and have shown previously that images obtained from two different protocols could be combined for analysis [8].

## Conclusions

In conclusion, these preliminary findings suggest that carrying the *BDNF* p.Met66 allele when exposed to early life adversity may not be associated with differential brain volumes in adolescents with AUD. Other genetic contributors should be investigated in future work.

## Abbreviations

AUD: Alcohol Use Disorders
*BDNF*: *Brain derived Neurotrophic Factor*
CPGR: Centre for Proteomic and Genomic Research
CSF: Cerebrospinal Fluid
CTQ: Childhood Trauma Questionnaire
CTQ-SF: Childhood Trauma Questionnaire- Short Form
DLPFC: Dorsolateral Prefrontal Cortex
FWE: Family-Wise Error
GM: Grey Matter
HC: Healthy Controls
HWE: Hardy-Weinberg Equilibrium
K-SADS-PL: Schedule for Affective Disorders and Schizophrenia for school-aged children Lifetime Version
MNI: Montreal Neurological Institute
MRI: Magnetic Resonance Images
Met: Methioinine
PFC: Prefrontal Cortex
ROI: Region of Interest
TLFB: Timeline Followback
TMV: Total Matter Volume
Val: Valine
VBM: Voxel Based Morphometry
WM: White Matter
